# Nomogram to predict survival of patients with advanced and metastatic pancreatic Cancer

**DOI:** 10.1186/s12885-021-08943-w

**Published:** 2021-11-15

**Authors:** G. C. Deng, Y. Lv, H. Yan, D. C. Sun, T. T. Qu, Y. T. Pan, Q. L. Han, G. H. Dai

**Affiliations:** 1 School of Medicine, Nankai University, Tianjin, China; 2 Senior Department of Oncology, The Fifth Medical Center of PLA General Hospital, Beijing, China

**Keywords:** Nomogram, Pancreatic cancer, Prognosis, Laboratory parameters, Clinicopathological factors

## Abstract

**Background:**

Nomograms are rarely employed to estimate the survival of patients with advanced and metastatic pancreatic cancer (PC). Herein, we developed a comprehensive approach to using a nomogram to predict survival probability in patients with advanced and metastatic PC.

**Methods**: A total of 323 patients with advanced and metastatic PC were identified from the Chinese People’s Liberation Army (PLA) General Hospital. A baseline nomogram was constructed using baseline variables of 323 patients. Additionally, 233 patients, whose tumors showed initial responses to first-line chemotherapy, were enrolled in the chemotherapy response-based model. 128 patients and 108 patients with advanced and metastatic PC from January 2019 to April 2021 were selected for external validating baseline model and chemotherapy response-based model. The 1-year and 2-year survival probability was evaluated using multivariate COX regression models. The discrimination and calibration capacity of the nomograms were assessed using C-statistic and calibration plots. The predictive accuracy and net benefit of the nomograms were evaluated using ROC curve and DCA, respectively.

**Results:**

In the baseline model, six variables (gender, KPS, baseline TB, baseline N, baseline WBC and baseline CA19–9) were used in the final model. In the chemotherapy response-based model, nine variables (KPS, gender, ascites, baseline N, baseline CA 19–9, baseline CEA, change in CA 19–9 level at week, change in CEA level at week and initial response to chemotherapy) were included in the final model. The C-statistics of the baseline nomogram and the chemotherapy response-based nomogram were 0.67 (95% CI, 0.62–0.71) and 0.74 (95% CI, 0.69–0.77), respectively.

**Conclusion:**

These nomograms were constructed to predict the survival probability of patients of advanced and metastatic PC. The baseline model and chemotherapy response-based model performed well in survival prediction.

**Supplementary Information:**

The online version contains supplementary material available at 10.1186/s12885-021-08943-w.

## Introduction

Pancreatic cancer (PC), a malignancy of the digestive system, shows high mortality. Indeed, PC is the seventh leading cause of cancer-related deaths worldwide [1]. In China, PC is the sixth leading cause of cancer-related mortality [2]. Currently, the 5-year survival rate of patients with PC is below 7% [3]. Comprehensive chemotherapy-based treatment regimens are the primary choice for patients with advanced or metastatic PC. The preferred chemotherapy regimens recommended by the guidelines of the National Comprehensive Cancer Network (NCCN) include FOLFIRINOX (5-FU), leucovorin, irinotecan, and oxaliplatin, or gemcitabine plus albumin-bound paclitaxel for patients with good performance status; gemcitabine monotherapy or 5-FU/capecitabine is used for patients with poor performance status [4]. The median overall survival time of patients receiving these chemotherapeutic regimens is currently less than one year [5].

The effects of chemotherapy vary in patients with advanced or metastatic PC; therefore, evaluating the prognosis of patients under different conditions is essential for both patients and oncologists. Previous studies have shown that certain risk factors are associated with survival [6–9]. Most predictive models can select some of the individual predictive factors, but cannot calculate survival probability [10–12], which is important to both patients and physicians.

Nomograms, employed to evaluate the survival probability of patients using a specific scoring system [13, 14], have been used to predict survival rates in patients with different cancers [15, 16]. However, few studies have used nomograms to predict the survival rate of patients with advanced or metastatic PC [17, 18]. Most models are constructed using baseline clinicopathological factors [19]. However, patient responses and sensitivity to chemotherapy are also crucial for constructing predictive models for patients undergoing chemotherapy.

In this study, we aimed to develop a baseline model, constructed using baseline factors, and a chemotherapy response-based model, constructed using factors involved in the initial response to chemotherapy and changes in the expression levels of tumor markers after two cycles of chemotherapy.

## Patients and methods

### Patient population

This study included patients with advanced and metastatic PC who underwent first-line chemotherapy from January 2010 to December 2018 at the Chinese People’s Liberation Army (PLA) General Hospital. Follow-up evaluations were performed every 6 months by telephone or by evaluation of medical records.

The inclusion criteria were as follows: (1) diagnosis was confirmed by histopathology or cytological evaluation (2); Karnofsky performance status (KPS) score ≥ 70 (3); no previous treatment using first-line chemotherapy (3); evaluation using computed tomography (CT) was conducted before the start of first-line chemotherapy (4); baseline information was collected before first-line chemotherapy (5); availability of CT scans at 6-weeks (before the third cycle of chemotherapy) (6); laboratory factors at 6 weeks, obtained before the third cycle of chemotherapy (7); having explicit terminal status.

From January 2019 to April 2021, an independent cohort of patients with advanced and metastatic PC who underwent first-line chemotherapy was prospectively studied, using the same inclusion and exclusion criteria. This independent group was used for validation cohort of this study.

In the train group, according to these criteria, 323 patients were selected into the group used to construct the baseline model. Among these patients, 233 patients, whose tumors had shown initial responses to first-line chemotherapy, were enrolled into the group used to construct the chemotherapy response-based model; these patients had been evaluated using CT scans before the third cycle of chemotherapy. In the validation group, 128 patients and 108 patients were selected for validating baseline model and chemotherapy response-based model. The time point of 6 weeks from the beginning of first-line chemotherapy was selected because chemotherapy regimens were administered using a 3-week cycle, and the evaluation was conducted before the start of third-cycle chemotherapy. Our present study included 134 patients in the train group, treated with nab-paclitaxel plus S-1 regimen in a NPSPAC clinical trial; this was a single-arm, single-center, phase II trial conducted at the Chinese People’s Liberation Army (PLA) General Hospital (https://clinicaltrials.gov/ number, NCT02124317).

### Clinicopathological variables

The following demographic and clinicopathological variables were collected: gender, age, body mass index (BMI), KPS, smoking status, use of alcohol, diabetes, jaundice, ascites, metastatic sites, total number of organs with metastases, and location of the primary tumor. The following laboratory values were collected: white blood cell (WBC), platelet (PLT), and neutrophil (N) count; and the levels of albumin (Alb), lactate dehydrogenase (LDH), total bilirubin (TB), serum carcinoembryonic antigen (CEA), and serum carbohydrate antigen 19–9 (CA 19–9). We also examined the levels of serum albumin, LDH, total bilirubin, CEA, and CA 19–9 at the 6-week time point. Cancerous lesions were assessed using CT scans before the first cycle, and after two cycles, of chemotherapy. Efficacy of chemotherapy was evaluated using Response Evaluation Criteria In Solid Tumors (RECIST, version 1.0), and patients were classified into the progressive disease (PD) group or non-progressive disease (non-PD) group according to tumor responses. Changes in tumor marker levels at 6 weeks were defined as (value measured at 6 weeks minus baseline value) divided by (baseline value); for example, a change in LDH value at 6 weeks = ([LDH at week 6] - [LDH at baseline]) / (LDH at baseline). Based on the obtained values, patients were divided into two categories: the value of ≥0 was defined as the no change and increase group, while the value of < 0 was defined as the decrease group.

### Statistical analysis

Continuous predictors were expressed using medians with interquartile ranges (IQRs), and categorical predictors were described using counts and proportions. Continuous variables (i.e., age) were categorized into two groups according to their median levels. Correlation of variables was evaluated using a correlation matrix. Overall survival (OS) was defined as the time interval beginning at the date of commencement of first-cycle chemotherapy to the date of death or final follow-up time. We firstly utilized univariate Cox regression to screen for survival related variables. Then, we used backward stepwise selection with Akaike information criterion (AIC) to further select variables. Based on the results of COX multivariate analysis, statistically significant variables were enrolled into the nomograms to predict the probability of survival using the rms package in R. Each nomogram was based on proportionally converting the regression coefficient of each independent risk factor in multivariate COX regression to a number on a 0- to 100-point scale. The points for each independent variable were added together to derive the total-point score for the predicted probability.

Performance of the predictive model was evaluated using a concordance statistic (C statistic) and calibration. The C-statistic is equal to the area under the receiver operating characteristic (ROC) curve. A C-statistic value of 0.5 indicates no predictive discrimination, while that of 1.0 indicates perfect separation of patients with different outcomes. Calibration with 1000 bootstrap samples to reduce overfitting was estimated by calibration plot. In a perfectly calibrated model, the prediction curve can coincide with the 45-degree diagonal line. The accuracy of the predictive model was evaluated by the ROC curve using the timeROC package in R. The net benefit of the model was assessed using decision curve analysis (DCA). We calculated the total scores of patients predicted by the nomograms, used X-tile version 3.6.1 (Yale University, New Haven, CT, USA) to determine the optimal cut-off value for stratifying patients, and performed Kaplan-Meier survival analysis (SPSS 26.0). All statistical analyses were performed in R (version 3.6.3), SPSS26.0, X-tile (version 3.6.1). *p* < 0.05 was considered statistically significant.

## Results

### Clinical characteristics of the patients

Among the 323 patients, median overall survival (mOS) was 10.6 months (95% CI: 9.7–11.7). The 1-year survival rate was 42.1% and 2-year survival rate was 12.2%.

**Fig. 1:**
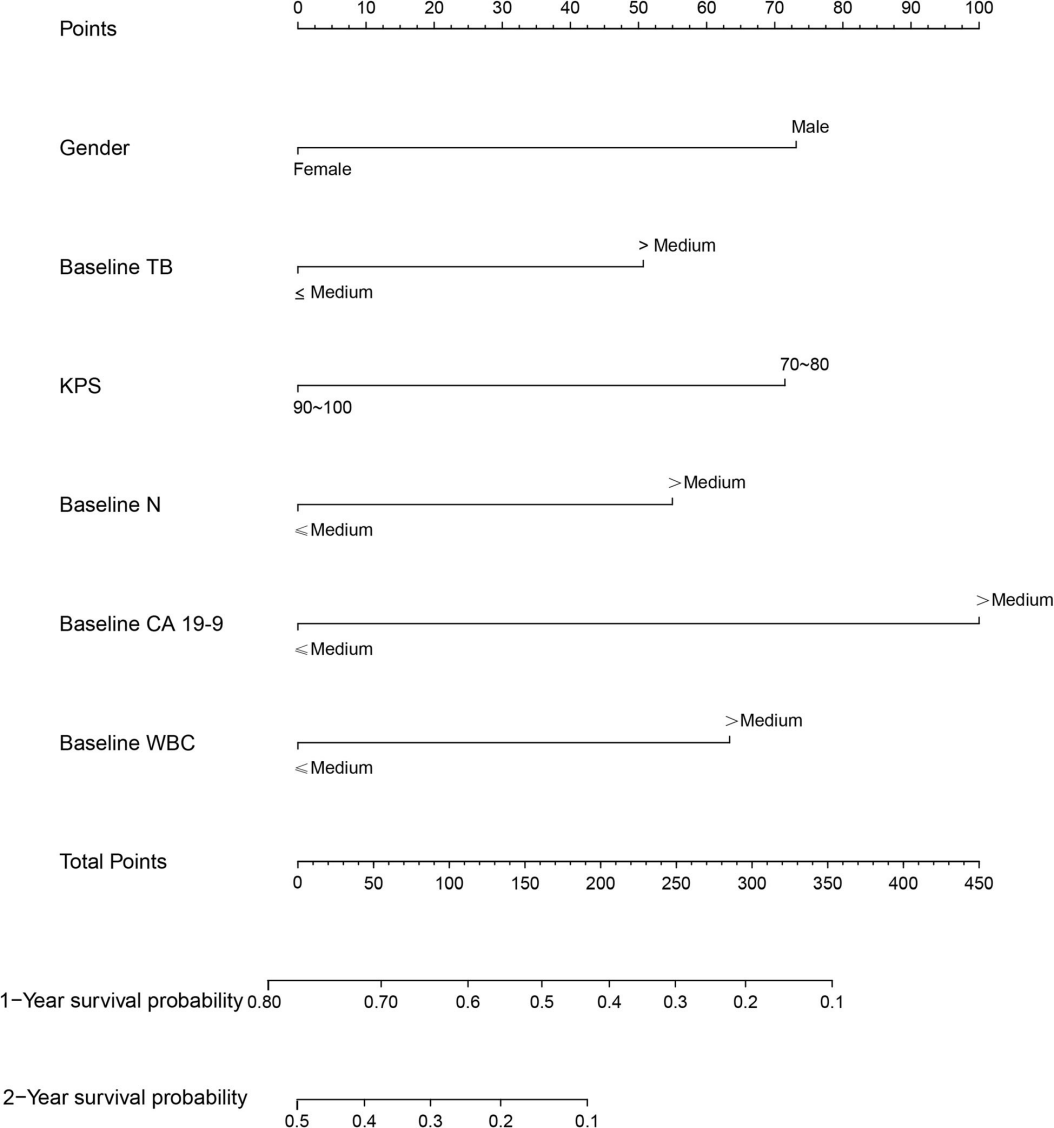
Baseline nomogram, used to predict 1-year and 2-year survival rate in patients with advanced and metastatic pancreatic cancer, created using four independent prognostic factors

In the train cohort, patient clinical characteristics in the baseline and chemotherapy response-based models are summarized in Table 1 and Table S1(Supplement). For continuous predictors, median levels with interquartile ranges (IQRs) are provided in Table S2 (Supplement). The patient clinical characteristics of validation cohort are listed in TableS3 and TablsS4 (Supplement). Median levels of the continuous variables were close in the two models. Data distribution was similar between the baseline model and chemotherapy response-based model. In the chemotherapy response-based model, 134 (57.5%) patients were treated with nab-paclitaxel plus S1 chemotherapy regimen, 31 (13.3%) patients were treated with gemcitabine monotherapy, 16 (6.9%) patients were treated with nab-paclitaxel plus gemcitabine, 41 (17.6%) patients were treated with gemcitabine-based combination chemotherapy, 4 (1.7%) patients were treated with S1 monotherapy, and 7 (3.0%) patients were treated with nab-paclitaxel monotherapy; 55 (23.6%) patients showed progressive disease at the first evaluation after the second cycle of chemotherapy. Survival curves of different first-line chemotherapy regimens were shown in TableS5 and Fig1S A-F (Supplement). The median overall survival of patients treated with nab-paclitaxel plus S1 was greater than gemcitabine monotherapy (9.92 months vs 6.18 months, *p* = 0.004). The effect of gemcitabine-based combination was superior to gemcitabine monotherapy (11.04 months vs 6.18 months, *p* = 0.019).

**Table 1:** Patient characteristics

Characteristic	Baseline model	Chemotherapy response-based model
	N Percent (%)	N Percent (%)
**Gender**		
Female	125 (38.7)	89 (38.2)
Male	198 (61.3)	144 (61.9)
**Age (year)**		
≤ 56	169 (52.3)	129 (55.4)
>56	154 (47.7)	104 (44.6)
**KPS**		
70 ~ 80	95 (29.4)	86 (36.9)
90 ~ 100	228 (70.6)	147 (63.1)
**Number of organ metastases**		
0	55 (17.0)	33 (14.2)
1	195 (60.4)	145 (62.2)
≥ 2	73 (22.6)	55 (23.6)
**Liver metastases**		
Absent	74 (22.9)	48 (20.6)
Present	249 (77.1)	185 (79.4)
**Baseline WBC (×1000/mm3)**		
≤ 6.39	162 (50.2)	113 (48.5)
> 6.39	161 (49.8)	120 (51.5)
**Baseline N**		
≤ 0.665	162 (50.2)	113 (48.5)
> 0.665	161 (49.8)	120 (51.5)
**Baseline LDH**		
≤ 159	155 (48.0)	116 (49.8)
> 159	168 (52.0)	117 (50.2)
**Baseline CEA**		
≤ 7.32	161 (49.8)	115 (49.4)
> 7.32	162 (50.2)	118 (50.6)
**Baseline CA 19–9**		
≤ 1453	162 (50.2)	112 (48.1)
> 1453	161 (49.8)	121 (51.9)
**Change in LDH level at week 6**	**NI**	
Decrease		80 (34.3)
No change or increase		153 (65.7)
**Change in CEA level at week 6**	**NI**	
Decrease		93 (39.9)
No change or increase		140 (60.1)
**Change in CA 19–9 level at week 6**	**NI**	
Decrease		151 (64.8)
No change or increase		82 (35.2)
**LDH at week 6**	**NI**	
≤ 170.7		113 (48.5)
> 170.7		120 (51.5)
**CEA at week 6**	**NI**	
≤ 7.97		117 (50.2)
> 7.97		116 (49.8)
**CA 19–9 at week 6**	**NI**	
≤ 790.7		114 (48.9)
> 790.7		119 (51.1)
**Alb level at week 6**	**NI**	
≤ 39.0		117 (50.2)
> 39.0		116 (49.8)
**Initial response to chemotherapy**	**NI**	
PD		55 (23.6)
Non-PD		178 (76.4)

### Development of the nomogram prognostic model

**Fig. 2:**
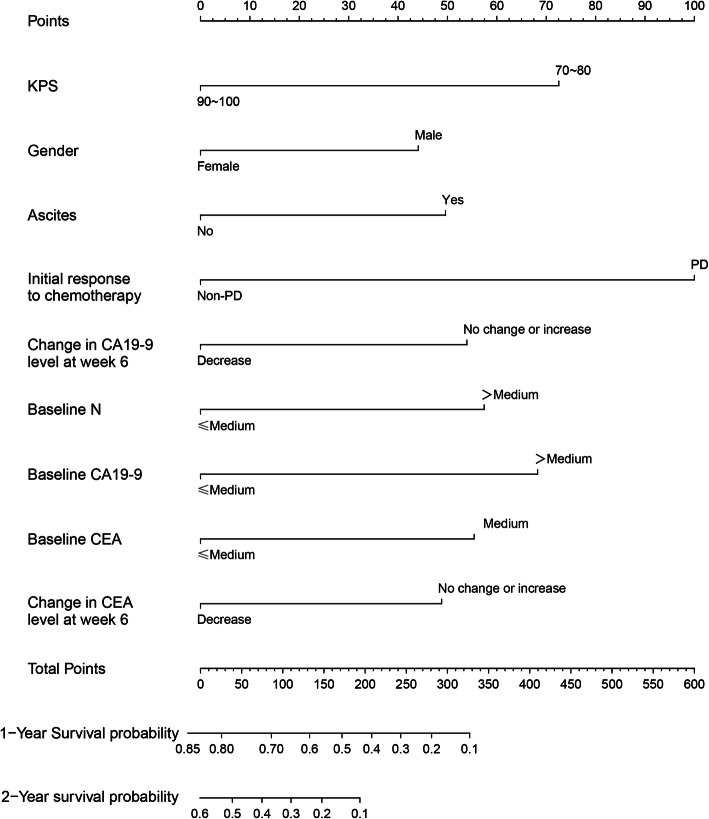
Chemotherapy response-based nomogram, used to predict 1-year and 2-year survival rate in patients with advanced and metastatic pancreatic cancer, created using five independent prognostic factors

All demographic and clinicopathological variables, as well as tumor markers, were selected as candidate factors for the development of the prediction model. In the baseline model, twelve variables were selected for multivariable analysis in univariate survival analysis (Table 2 and Table S6, Supplement). Correlation analysis between twelve variables shown FigS2 (Supplement). In multivariable survival analysis, we used stepwise AIC backward regression to identify the six factors for the final prediction model: gender, KPS, baseline TB, baseline N, baseline WBC and baseline CA19–9 (Table 3).

**Table 2:** Results of univariate survival analysis

Characteristic	Baseline model (***N*** = 323)			Chemotherapy response-based model (***N*** = 233)		
	HR	95% CI	*p* value	HR	95% CI	*p* value
**Gender** (female vs male)	1.45	1.10–1.92	**0.008**	1.49	1.09–2.05	**0.011**
**Age** (≤56 vs > 56)	1.01	1.00–1.03	**0.012**	1.22	0.90–1.66	0.199
**KPS** (70 ~ 80 vs 90 ~ 100)	0.57	0.43–0.77	**< 0.001**	0.45	0.33–0.63	**< 0.001**
**Smoke** (No vs Yes)	1.32	1.00–1.74	**0.047**	1.41	1.03–1.94	**0.034**
**Ascites** (No vs Yes)	1.46	1.08–1.96	**0.012**	1.54	1.11–2.16	**0.011**
**Baseline WBC** (≤ 6.39 vs > 6.39)	1.63	1.25–2.13	**< 0.001**	1.41	1.04–1.91	**0.027**
**Baseline N** (≤ 0.665 vs > 0.665)	1.60	1.23–2.09	**0.001**	1.68	1.24–2.29	**0.001**
**Baseline LDH** (≤159 vs > 159)	1.38	1.06–1.80	**0.018**	1.14	0.84–1.54	0.402
**Baseline TB** (≤11.4 vs>11.4)	1.45	1.11–1.89	**0.006**	1.43	1.06–1.94	**0.021**
**Baseline Alb** (≤ 40.5 vs > 40.5)	0.76	0.58–1.00	**0.049**	0.81	0.59–1.09	0.166
**Baseline CEA** (≤7.32 vs > 7.32)	1.47	1.13–1.92	**0.005**	1.44	1.06–1.95	**0.019**
**Baseline CA 19–9** (≤1453 vs > 1453)	1.81	1.38–2.37	**< 0.001**	1.72	1.26–2.34	**0.001**
**Change in CEA level at week 6**		**NI**				
Decrease					Ref	
No change or increase				1.39	1.02–1.91	**0.039**
**Change in CA19–9 level at week 6**		**NI**				
Decrease					Ref	
No change or increase				1.69	1.23–2.31	**0.001**
**Albumin at week 6** (≤39.0 vs>39.0)		**NI**		0.67	0.49–0.91	**0.010**
**TB at week 6** (≤11.3 vs>11.3)		**NI**		1.36	1.01–1.85	**0.047**
**CEA at week 6** (≤7.97 vs>7.97)		**NI**		1.58	1.17–2.14	**0.003**
**CA 19–9 at week 6** (≤790.7 vs>790.7)		**NI**		1.99	1.46–2.73	**< 0.001**
**Initial response to chemotherapy**		**NI**				
Non-PD					Ref	
PD				3.07	2.17–4.35	**< 0.001**

**Table 3:** Results of multivariate survival analysis

Characteristic	Baseline model (***N***=323)			Chemotherapy response-based model (***N***=233)		
	HR	95% CI	*p* value	HR	95% CI	*p* value
**Gender** (female vs male)	1.71	1.33-2.19	**<0.001**	1.40	1.01-1.94	**0.039**
**KPS** (70~80 vs 90~100)	0.68	0.49-0.94	**0.018**	0.57	0.40-0.80	**0.001**
**Ascites**		**NI**		1.47	1.04-2.08	**0.031**
**Baseline WBC**	1.55	1.17-2.06	**0.002**		**NI**	
**Baseline N**	1.35	1.03-1.78	**0.032**	1.56	1.14-2.13	**0.005**
**Baseline TB**	1.44	1.09-1.91	**0.012**		**NI**	
**Baseline CA 19-9**	1.87	1.41-2.47	**<0.001**	1.70	1.21-2.38	**0.002**
**Baseline CEA**		**NI**		1.54	1.09-2.19	**0.017**
**Change in CA19–9 level at week 6**		**NI**				
Decrease					**Ref**	
No change or increase				1.51	1.06-2.14	**0.017**
**Change in CEA level at week 6**		**NI**				
Decrease					**Ref**	
No change or increase				1.48	1.02-2.14	**0.039**
**Initial response to chemotherapy**		**NI**				
PD					**Ref**	
Non-PD				0.46	0.31-0.69	**<0.001**

**Fig. 3:**
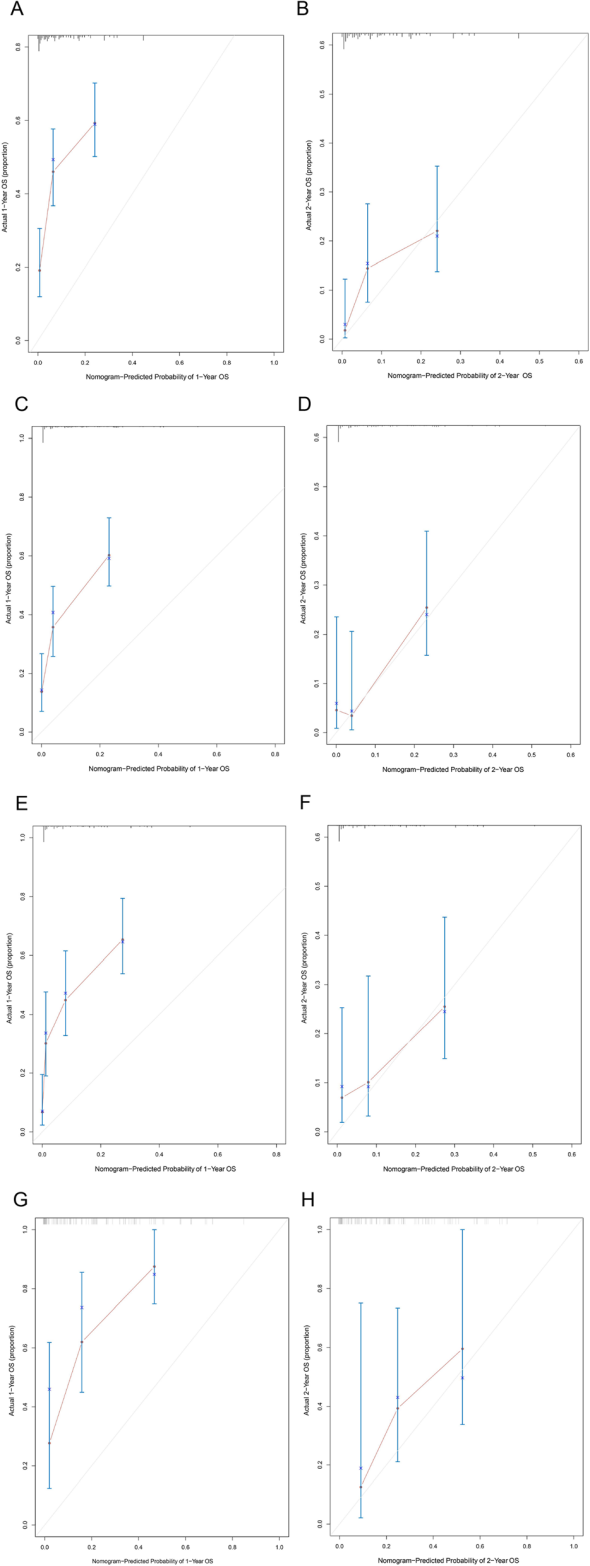
Calibration plots of the two nomogram models comparing predicted probabilities against actual 1-year and 2-year survival rate. (**A**) Calibration plot of 1-year survival predictive in the baseline nomogram; (**B**) Calibration plot of 2-year survival predictive in the baseline nomogram; (**C**) Calibration plot of 1-year survival predictive in the chemotherapy response-based nomogram; (**D**) Calibration plot of 2-year survival predictive in the chemotherapy response-based nomogram; (**E**) Calibration plot of 1-year survival predictive in external baseline validation group; (**F**) Calibration plot of 2-year survival predictive in external baseline validation group;(**G**) Calibration plot of 1-year survival predictive in external chemotherapy validation group;(**H**) Calibration plot of 2-year survival predictive in external chemotherapy validation group

Similarly, in the chemotherapy response-based model, 16 variables showing statistical significance in univariate analysis were included in the correlation analysis. We excluded two variables (CA 19–9 level at 6 week and CEA level at 6 week) to decrease the influence on survival by multicollinearity (FigS3, Supplement). We used stepwise AIC backward regression to identify the nine factors for the final nomogram model: KPS, gender, ascites, baseline N, baseline CA 19–9, baseline CEA, change in CA 19–9 level at week, change in CEA level at week and initial response to chemotherapy (Table 3). Our results indicate that these nomograms can be used to evaluate the survival probability in patients with advanced and metastatic PC (Figs. 1 and 2).

### Nomogram validation

**Fig. 4:**
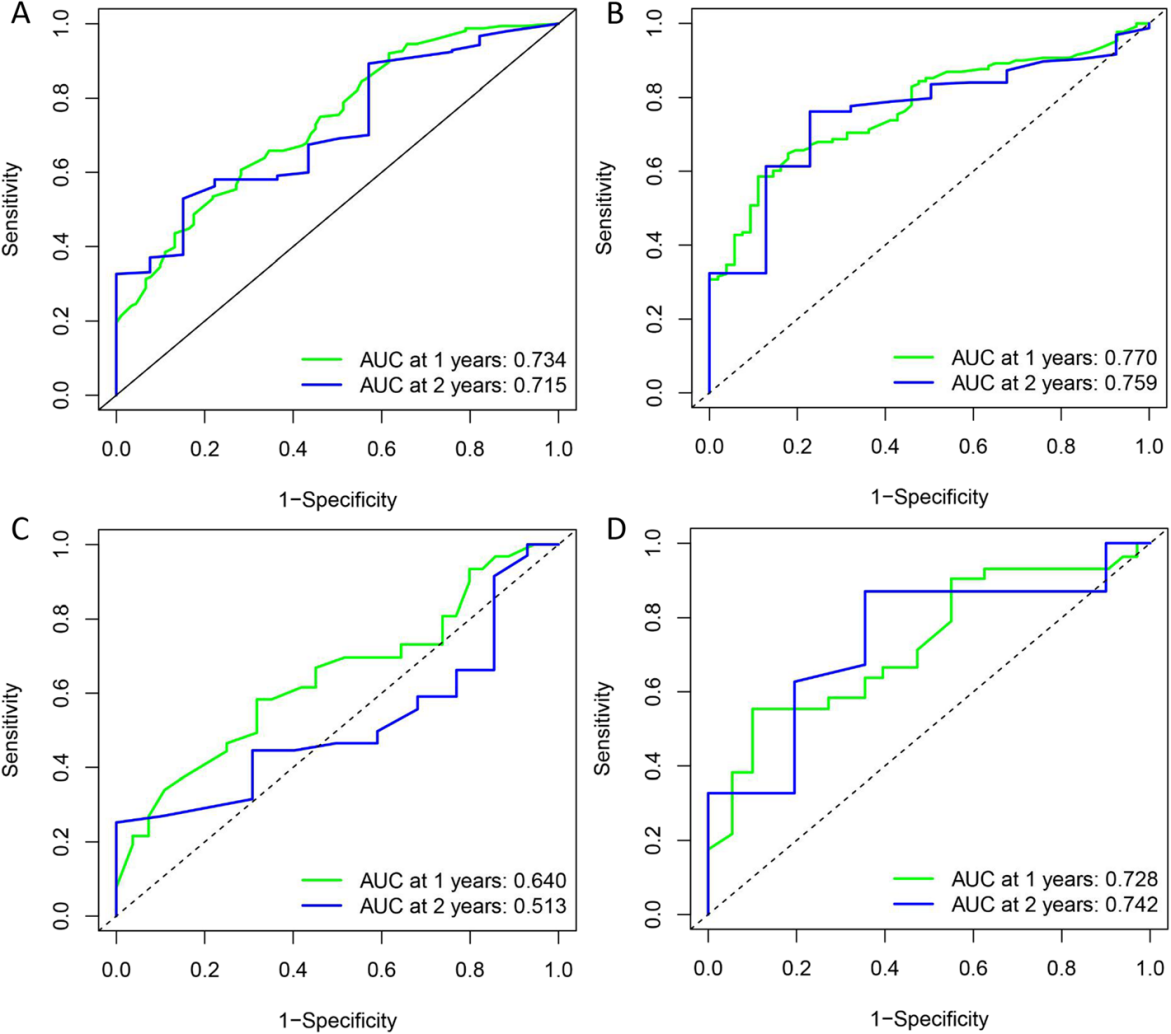
Time-dependent receiver operating characteristic (ROC) curve was used to analyze the value of the nomograms in predicting 1-year and 2-year survival. (**A**) ROC curve of 1-year and 2-year survival predictive of baseline model in the train group; (**B**) ROC curve of 1-year and 2-year survival predictive of chemotherapy response-based model in the train group; (**C**)ROC curve of 1-year and 2-year survival predictive of baseline model in the validation group; (**D**) ROC curve of 1-year and 2-year survival predictive in the chemotherapy response-based model in the validation group

For the baseline and chemotherapy response-based models, C-statistic, confirmed using 1000 bootstrap validation, were 0.67 (95% CI, 0.62–0.71) and 0.74 (95% CI, 0.69–0.77), respectively. Calibration curves also indicated good agreement between prediction and observation in the baseline and chemotherapy response-based nomogram models (Fig. 3A, B). In validation group, calibration curves demonstrated the good performance of two nomogram models (Fig. 3C-H). The accuracy of baseline and chemotherapy response-based nomogram model performed better in 2-year survival prediction than 1-year survival prediction. Predictive accuracy of the models with respect to individual and combined factors was compared using the ROC curves (Fig. 4A-D). Both in the train and validation group, the AUC of ROC curves could demonstrate the reliable of baseline and chemotherapy response-based models.

### Clinical use

**Fig. 5:**
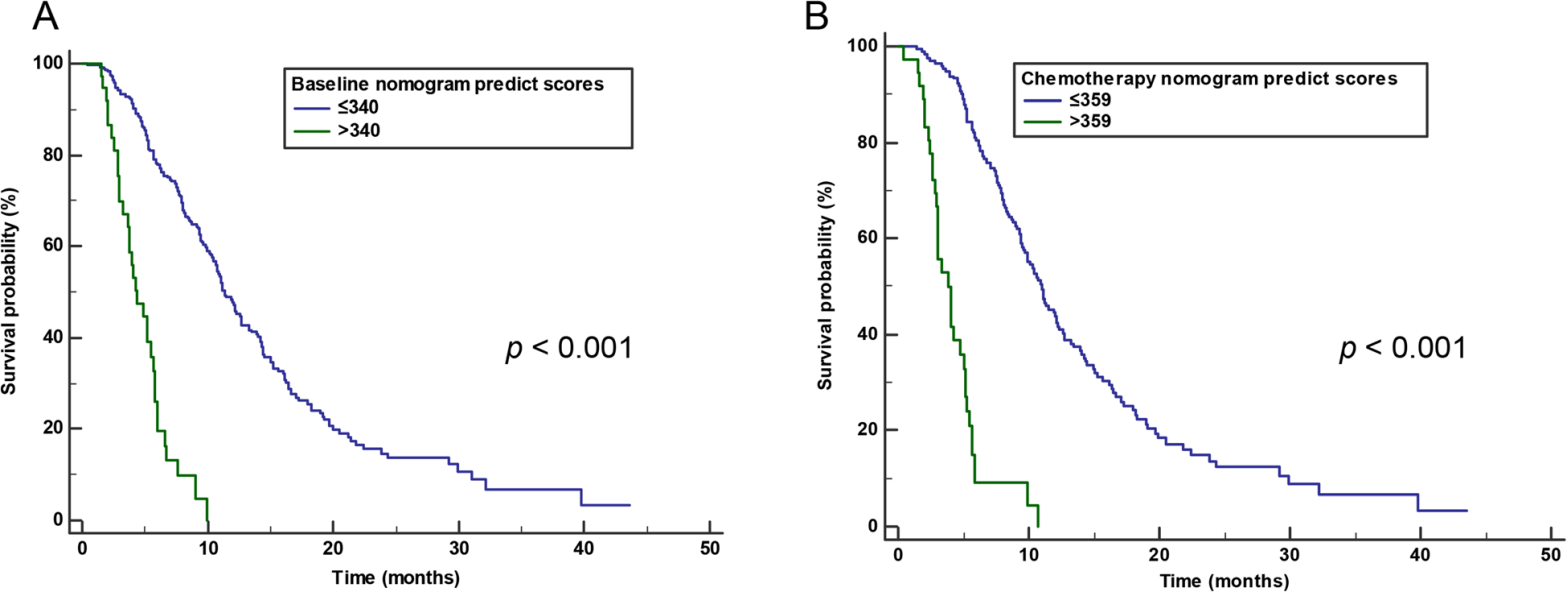
Kaplan-Meier curves for patients stratified by the nomogram predictive scores. (**A**) Kaplan-Meier curves for patients evaluated using the baseline nomogram; (**B**) Kaplan-Meier curves for patients evaluated using the chemotherapy response-based nomogram

We used X-tile to determine the optimal cut-off value in stratifying patients based on total scores predicted by the nomograms; then, we plotted the Kaplan-Meier survival curves (Fig. 5A, B). The baseline model stratified at 340 scores and indicated increased survival time for patients with a total score higher than 340 (HR, 6.03; 95% CI, 3.99–9.10; *p* < 0.001). The chemotherapy response-based model stratified at 359 scores and indicated increased survival time for patients with scores higher than 359 (HR, 7.94; 95% CI, 5.17–12.19; *p* < 0.001).

**Fig. 6:**
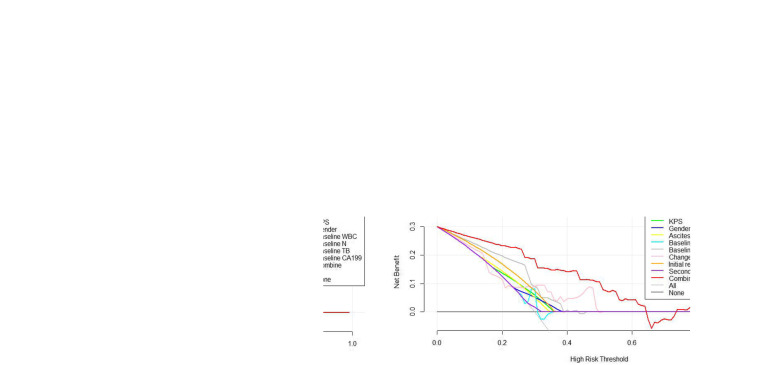
Decision curve analysis (DCA) of the nomograms in prediction of 1-year and 2-year survival. The dark solid line represents the assumption that no patient survived beyond 1 or 2 year. The gray solid line represents the assumption that all patients survived in 1 or 2 year. The red solid line represents the combined nomogram. (**A**) DCA of 1-year survival predictive in the baseline nomogram; (**B**) DCA of 2-year survival predictive in the baseline nomogram; (**C**) DCA of 1-year survival predictive chemotherapy response-based nomogram; (**D**) DCA of 2-year survival predictive chemotherapy response-based nomogram

We also used decision curve analysis (DCA) to evaluate the net benefit and clinical application value of our nomogram models. In the two nomogram models, the combined predictive models showed better clinical utility than the predictive value of any single variable (Fig. 6A-D).

## Discussion

In this study, we constructed two nomogram models that can be used to predict survival probability in patients with advanced and metastatic PC treated with first-line chemotherapy. Performance of the nomograms was rigorously assessed and internally validated. As shown by clinical trials, survival time of patients with advanced PC has gradually increased, but median overall survival remains less than 1 year with palliative chemotherapy [5]. Data on patient survival, estimated by prognostic models, can be used by physicians to prescribe suitable treatment and adjust therapies in a timely manner.

In our study, we used nomograms to predict the survival probability of patients with advanced PC. Our baseline nomogram, constructed using baseline clinical factors easily obtained before chemotherapy, showed good performance. This nomogram will aid physicians in making a preliminary survival assessment at the time of diagnosis and in prescribing appropriate dosage regimens. The chemotherapy response-based model was constructed after patients had undergone two cycles of chemotherapy. This model used factors such as initial response to chemotherapy and changes in the expression levels of tumor markers compared with those at baseline. This nomogram can be used to guide physicians in their decisions on whether to adjust treatment strategies.

In this study, we collected a wide array of variables previously reported to be associated with the prognosis of patients with PC [20, 21]. Some of the risk factors associated with PC were also included in this study [22, 23]. Previous studies have shown that changes in the levels of tumor markers are associated with survival of patients with advanced PC [24, 25]. For comprehensive assessment of patient survival, we collected more factors than the numbers used in other studies. However, we did not incorporate all the variables into our model. After univariate analysis, we first used numerous association analyses to evaluate all the variables to avoid collinearity interference between variables. We then eliminated the variables having high correlation coefficients (*r* > 0.5, *p* < 0.05). These variables have also been repeatedly validated in different studies, which helped us in locating and selecting pertinent information from large repositories of clinical data [18, 21].

Our baseline and chemotherapy response-based models demonstrated good discriminative ability, with C-statistic of 0.67 and 0.74, respectively. The baseline model was similar to those constructed in previous studies [21, 26]. In the two models constructed in our present study, baseline CA 19–9 level was an important factor for predicting the prognosis of patients with advanced PC. Previous studies have also shown that baseline CA 19–9 level is important in the construction of predictive models [20, 21, 27]. Because survival can be affected by the treatment process, we incorporated additional factors into our chemotherapy response-based model to improve the prognostic ability of the model; these factors included initial response to chemotherapy and expression levels of important laboratory markers. We found that in initial response to chemotherapy and changes in the levels of laboratory markers at 6 weeks were associated with survival probability, as shown by univariate analyses. We also performed Kaplan-Meier survival analyses of different first-line chemotherapy regimens. The results showed that the median overall survival of gemcitabine-based combination chemotherapy and nab-paclitaxel plus S1 was better than gemcitabine monotherapy with statistic significant (TableS5 and Fig1S A-F Supplement). Our team retrospectively analyzed multicenter first-line chemotherapy regimens of advanced pancreatic cancer, the results showed that nab-paclitaxel plus S1 was not inferior to nab-paclitaxel plus gemcitabine or FOLFIRINOX (5-fluorouracil, leucovorin, irinotecan, and oxaliplatin) [28]. However, the final model did not incorporate chemotherapy regimens or laboratory markers levels at 6 weeks, unless these changes were shown statistically significant by multivariate analysis. Our multivariate analysis indicated that the initial response to chemotherapy also was powerful in predicting patient survival. The initial response to chemotherapy can also be used to predict the response of other tumors to the chemotherapy being used [16, 29].

Our study had several limitations. Because it was a retrospective study, it may have been subject to biases. Additionally, our data were collected at a single center; therefore, in our future studies, we will obtain and combine data from multi-center databases to increase the credibility of our results. Finally, the patients evaluated in our study may have been subject to different subsequent treatment regimens. This factor, however, was not addressed in detail in our present study.

## Conclusions

In this study, we used different clinical factors to construct nomograms that can be utilized to evaluate the survival probability of patients with advanced PC undergoing first-line chemotherapy. The baseline and chemotherapy response-based models developed in our present study showed good fit. Information obtained using these nomogram models can be used to assist clinicians in the selection and adjustment of treatment strategies. In our future studies, we will optimize these predictive models using multi-center data.

## Supplementary Information

**Additional file 1: Table S1.** Patient characteristics of train groups (supplement data). **Table S2.** Patient Characteristics of train groups. **Table S3.** Patient characteristics of the validation groups. **Table S4.** Patient characteristics of validation groups. **Table S5.** Survival analysis of first-line chemotherapy regimens in train group. **Table S6.** Results of univariate survival analysis in train cohort (supplement data). **Fig. S1.** Kaplan-Meier survival curves of different chemotherapy regimens. A. Gem vs AS. B. Gem vs Gem-based. C. Gem vs AG. D. AS vs Gem-based. E. AS vs AG. F. Survival analysis of all chemotherapy regimens. Abbreviation: Gem: gemcitabine monotherapy; Gem-based: gemcitabine-based combination chemotherapy; AS: nab-paclitaxel plus S1; AG: nab-paclitaxel plus gemcitabine. **Fig. S2.** Correlation analysis between twelve survival-related variables in baseline group. **Fig. S3.** Correlation analysis between eighteen survival-related variables in chemotherapy group.

## Data Availability

The data used to support the findings of this study are available from the corresponding author upon request.

## Abbreviations

KPS: Karnofsky performance status
CT: computed tomography
WBC: white blood cell
PLT: platelet
N: neutrophil
Alb: albumin
LDH: lactate dehydrogenase
TB: total bilirubin
CEA: carcinoembryonic antigen
CA 19–9: serum carbohydrate antigen 19–9
PD: progressive disease
IQRs: interquartile ranges
VIF: Variance inflation factor
OS: overall survival
AIC: Akaike information criterion
C-index: concordance index
ROC: receiver operating characteristic
DCA: decision curve analysis
AUC: area under the curve
