# Supplementary material for: Nomogram to predict survival of patients with advanced and metastatic pancreatic Cancer
Source: BMC Cancer. 2021 Nov 15;21:1227. doi: 10.1186/s12885-021-08943-w (PMC8594118; doi:10.1186/s12885-021-08943-w)
Supplement: Supplementary file 1 — Additional file 1: Table S1. Patient characteristics of train groups (supplement data). Table S2. Patient Characteristics of train groups. Table S3. Patient characteristics of the validation groups. Table S4. Patient characteristics of validation groups. Table S5. Survival analysis of first-line chemotherapy regimens in train group. Table S6. Results of univariate survival analysis in train cohort (supplement data). Fig. S1. Kaplan-Meier survival curves of different chemotherapy regimens. A. Gem vs AS. B. Gem vs Gem-based. C. Gem vs AG. D. AS vs Gem-based. E. AS vs AG. F. Survival analysis of all chemotherapy regimens. Abbreviation: Gem: gemcitabine monotherapy; Gem-based: gemcitabine-based combination chemotherapy; AS: nab-paclitaxel plus S1; AG: nab-paclitaxel plus gemcitabine. Fig. S2. Correlation analysis between twelve survival-related variables in baseline group. Fig. S3. Correlation analysis between eighteen survival-related variables in chemotherapy group. [file 12885_2021_8943_MOESM1_ESM.zip › Additional file1.docx]

**TableS1.** Patient characteristics of train groups (supplement data)

| **Characteristic** | **Baseline model** | **Chemotherapy response-based model** |
| --- | --- | --- |
|  | **N Percent (%)** | **N Percent (%)** |
| **BMI (kg/m^2^)** |  |  |
| ≤25 | 245 (75.9) | 168 (72.1） |
| ＞25 | 78 (24.1) | 65 (27.9） |
| **Smoking status** |  |  |
| Absent | 202 (62.5) | 148 (63.5） |
| Present | 121 (37.5) | 85 (36.5） |
| **Alcohol use** |  |  |
| Absent | 191 (59.1) | 135 (57.9） |
| Present | 132 (40.9) | 98 (42.1） |
| **Diabetes** |  |  |
| Absent | 255 (78.9) | 183 (78.5） |
| Present | 68 (21.1) | 50 (21.5） |
| **Jaundice** |  |  |
| Absent | 254 (78.6) | 185 (79.4） |
| Present | 69 (21.4) | 48 (20.6） |
| **Ascites** |  |  |
| Absent | 244 (75.5) | 174 (74.7） |
| Present | 79 (24.5) | 59 (25.3） |
| **Lung metastases** |  |  |
| Absent | 266 (82.4) | 190 (81.5） |
| Present | 57 (17.6) | 43 (18.5） |
| **Bone metastases** |  |  |
| Absent | 294 (91.0) | 213 (91.4） |
| Present | 29 (9.0) | 20 (8.6） |
| **Primary tumor location** |  |  |
| Head | 121 (37.5) | 86 (36.9） |
| Body-Tail | 202 (62.5) | 147 (63.1） |
| **Stage** |  |  |
| III | 55(17.0) | 33(14.2) |
| IV | 268(83.0) | 200(85.8) |
| **Baseline PLT (×1000/mm3)** |  |  |
| ≤Median | 162 (50.2) | 120 (51.5) |
| > Median | 161 (49.8) | 113 (48.5) |
| **Baseline Alb** |  |  |
| ≤Median | 164 (50.8) | 121 (51.9) |
| > Median | 159 (49.2) | 112 (48.1) |
| **Baseline TB** |  |  |
| ≤Median | 164 (50.8) | 121 (51.9) |
| > Median | 159 (49.2) | 112 (48.1) |
|  |  |  |

**(continue)**

| **Characteristic** | **Baseline model** | **Chemotherapy response-based model** |
| --- | --- | --- |
|  | **N Percent (%)** | **N Percent (%)** |
| **Change in Alb level at week 6** | **NI** |  |
| Decrease |  | 154 (66.1） |
| No change or increase |  | 79 (33.9） |
| **Change in TB level at week 6** | **NI** |  |
| Decrease |  | 138 (59.2) |
| No change or increase |  | 95 (40.8) |
| **TB at week 6** | **NI** |  |
| ≤Median |  | 122 (52.4) |
| > Median |  | 111 (47.6) |
| **Chemotherapy regimen** | **NI** |  |
| nab-paclitaxel plus S1 |  | 134 (57.5) |
| Non nab-paclitaxel plus S1 |  | 99 (42.5) |
| **Second-line chemotherapy** | **NI** |  |
| No |  | 117 (50.4) |
| Yes |  | 115 (49.6) |
| **Previous surgery** |  |  |
| No | 306 (94.7) | 221 (94.8) |
| Yes | 17 (5.3) | 12 (5.2) |

**TableS2.** Patient Characteristics of train groups

| **Characteristic** | **Baseline population** | **Chemotherapy population** |
| --- | --- | --- |
| **Age(year)** |  |  |
| Median | 56 | 56 |
| Interquartile ranges (IQR) | 50~62 | 49~61 |
| **Baseline WBC (×1000/mm3)** |  |  |
| Median | 6.34 | 6.39 |
| Interquartile ranges (IQR) | 5.16~7.79 | 5.18~7.77 |
| **Baseline PLT(×1000/mm3)** |  |  |
| Median | 199 | 198 |
| Interquartile ranges (IQR) | 150~258 | 148~238 |
| **Baseline N** |  |  |
| Median | 0.662 | 0.665 |
| Interquartile ranges (IQR) | 0.586~0.720 | 0.596~0.721 |
| **Baseline albumin** |  |  |
| Median | 40.6 | 40.5 |
| Interquartile ranges (IQR) | 37.5~43.6 | 37.3~43.4 |
| **Baseline LDH** |  |  |
| Median | 159 | 159 |
| Interquartile ranges (IQR) | 136.8~192.8 | 135.9~194.3 |
| **Baseline CEA** |  |  |
| Median | 7.06 | 7.32 |
| Interquartile ranges (IQR) | 2.56~23.6 | 2.34~25.25 |
| **Baseline CA199** |  |  |
| Median | 1081 | 1453 |
| Interquartile ranges (IQR) | 106.5~7039.0 | 136.2~8751.0 |
| **Baseline bilirubin** |  |  |
| Median | 11.3 | 11.4 |
| Interquartile ranges (IQR) | 8.5~15.2 | 8.7~15.7 |
| **Albumin level at week-6** | **NI** |  |
| Median |  | 39.0 |
| Interquartile ranges (IQR) |  | 35.9~41.3 |
| **LDH at week-6** | **NI** |  |
| Median |  | 170.7 |
| Interquartile ranges (IQR) |  | 149.9~209.7 |
| **CEA at week-6** | **NI** |  |
| Median |  | 7.97 |
| Interquartile ranges (IQR) |  | 3.19~21.25 |
| **CA199 at week-6** | **NI** |  |
| Median |  | 790.7 |
| Interquartile ranges (IQR) |  | 91.8~4535.5 |
| **Bilirubin at week-6** | **NI** |  |
| Median |  | 9.6 |
| Interquartile ranges (IQR) |  | 7.2~14.3 |

**TableS3.** Patient characteristics of the validation groups

| **Characteristic** | **Baseline model** | **Chemotherapy response-based model** |
| --- | --- | --- |
|  | **N Percent (%)** | **N Percent (%)** |
| **Gender** |  |  |
| Female | 42(32.8) | 37(34.3) |
| Male | 86(67.2) | 71(65.7) |
| **Age (year)** |  |  |
| ≤57 | 70(54.7) | 59(54.6) |
| ＞57 | 58(45.3) | 49(45.4) |
| **BMI (kg/m^2^)** |  |  |
| ≤22 | 63(49.2) | 54(50) |
| ＞22 | 65(50.8) | 54(50) |
| **KPS** |  |  |
| 70~80 | 50(39.0) | 33(30.6) |
| 90~100 | 78(61.0) | 75(69.4) |
| **Smoking status** |  |  |
| Absent | 83(64.8) | 71(65.7) |
| Present | 45(35.2) | 37(34.3) |
| **Alcohol use** |  |  |
| Absent | 71(55.5) | 61(56.5) |
| Present | 57(44.5) | 47(43.5) |
| **Diabetes** |  |  |
| Absent | 103(80.5) | 88(81.5) |
| Present | 25(19.5) | 20(18.5) |
| **Jaundice** |  |  |
| Absent | 97(75.8) | 83(76.9) |
| Present | 31(24.2) | 25(23.1) |
| **Ascites** |  |  |
| Absent | 102(79.7) | 86(79.6) |
| Present | 26(20.3) | 22(20.4) |
| **Number of** **organ metastases** |  |  |
| 0 | 29(22.7) | 21 |
| 1 | 79(61.7) | 69 |
| ≥2 | 20(15.6) | 18 |
| **Liver metastases** |  |  |
| Absent | 45(35.2) | 35 |
| Present | 83(64.8) | 73 |
| **Lung metastases** |  |  |
| Absent | 107(83.6) | 89 |
| Present | 21(16.4) | 19 |
| **Bone metastases** |  |  |
| Absent | 114(89.1) | 95 |
| Present | 14(10.9) | 13 |

(Continue)

| **Characteristic** | **Baseline model** | **Chemotherapy response-based model** |
| --- | --- | --- |
|  | **N Percent (%)** | **N Percent (%)** |
| **Primary tumor location** |  |  |
| Head | 54(42.2) | 44 |
| Body-Tail | 74(57.8) | 64 |
| **Baseline WBC (×1000/mm3)** |  |  |
| ≤Median | 64(50.0) | 54(50) |
| > Median | 64(50.0) | 54(50) |
| **Baseline PLT** |  |  |
| ≤Median | 64(50.0) | 55(50.9) |
| > Median | 64(50.0) | 53(49.1) |
| **Baseline N** |  |  |
| ≤Median | 64(50.0) | 54(50) |
| > Median | 64(50.0) | 54(50) |
| **Baseline LDH** |  |  |
| ≤Median | 64(50.0) | 54(50) |
| > Median | 64(50.0) | 54(50) |
| **Baseline Alb** |  |  |
| ≤Median | 64(50.0) | 55(50.9) |
| > Median | 64(50.0) | 53(49.1) |
| **Baseline TB** |  |  |
| ≤Median | 64(50.0) | 54(50) |
| > Median | 64(50.0) | 54(50) |
| **Baseline CA 19-9** |  |  |
| ≤Median | 64(50.0) | 54(50) |
| > Median | 64(50.0) | 54(50) |
| **Baseline CEA** |  |  |
| ≤Median | 64(50.0) | 54(50) |
| > Median | 64(50.0) | 54(50) |
| **Alb level at week 6** | **NI** |  |
| ≤Median |  | 57(52.8) |
| > Median |  | 51(47.2) |
| **TB at week 6** | **NI** |  |
| ≤Median |  | 54(50) |
| > Median |  | 54(50) |
| **LDH at week 6** | **NI** |  |
| ≤Median |  | 54(50) |
| > Median |  | 54(50) |
| **CEA at week 6** | **NI** |  |
| ≤Median |  | 54(50) |
| > Median |  | 54(50) |
| (Continue) | | |
| **Characteristic** | **Baseline model** | **Chemotherapy response-based model** |
|  | **N Percent (%)** | **N Percent (%)** |
| **CA 19-9 at week 6** | **NI** |  |
| ≤Median |  | 54(50) |
| > Median |  | 54(50) |
| **Change in LDH level at week 6** | **NI** |  |
| Decrease |  | 42(38.9) |
| No change or increase |  | 66(61.1) |
| **Change in CEA level at week 6** | **NI** |  |
| Decrease |  | 41(38.0) |
| No change or increase |  | 67(62.0) |
| **Change in Alb level at week 6** | **NI** |  |
| Decrease |  | 62(57.4) |
| No change or increase |  | 46(42.6) |
| **Change in TB level at week 6** | **NI** |  |
| Decrease |  | 65(60.2) |
| No change or increase |  | 43(39.8) |
| **Change in CA 19-9 level at week 6** | **NI** |  |
| Decrease |  | 70(64.8) |
| No change or increase |  | 38(35.2) |
| **Initial response to chemotherapy** | **NI** |  |
| PD |  | 16(14.8) |
| Non-PD |  | 92(85.2) |
| **Chemotherapy regimen** | **NI** |  |
| nab-paclitaxel plus S1 |  | 63(58.3) |
| Non nab-paclitaxel plus S1 |  | 45(41.7) |
| **Second-line chemotherapy** | **NI** |  |
| No |  | 92(85.2) |
| Yes |  | 16(14.8) |
| **Previous surgery** |  |  |
| No | 80(62.5) | 70(64.8) |
| Yes | 48(37.5) | 38(35.2) |
| **Stage** |  |  |
| III | 13(8.6) | 7(6) |
| IV | 115(89.8) | 101(94) |

**TableS4.** Patient characteristics of validation groups

| **Characteristic** | **Baseline population** | **Chemotherapy population** |
| --- | --- | --- |
| **Age(year)** |  |  |
| Median | 57 | 57 |
| Interquartile ranges (IQR) | 50-63 | 50-63 |
| **Baseline WBC (×1000/mmo3)** |  |  |
| Median | 5.88 | 5.94 |
| Interquartile ranges (IQR) | 4.65-7.48 | 4.78-7.41 |
| **Baseline PLT(×1000/mm3)** |  |  |
| Median | 191 | 192 |
| Interquartile ranges (IQR) | 152-245 | 152-247 |
| **Baseline N** |  |  |
| Median | 0.641 | 0.641 |
| Interquartile ranges (IQR) | 0.560-0.703 | 0.568-0.693 |
| **Baseline albumin** |  |  |
| Median | 39.9 | 40.3 |
| Interquartile ranges (IQR) | 38.4-42.8 | 38.7-40.3 |
| **Baseline LDH** |  |  |
| Median | 155.5 | 156.3 |
| Interquartile ranges (IQR) | 134.7-184.4 | 135.6-187.6 |
| **Baseline CEA** |  |  |
| Median | 4.69 | 5.26 |
| Interquartile ranges (IQR) | 2.68-4.69 | 2.86-14.1 |
| **Baseline CA199** |  |  |
| Median | 323.9 | 323.9 |
| Interquartile ranges (IQR) | 43.4-1333.5 | 51.3-1401.9 |
| **Baseline bilirubin** |  |  |
| Median |  | 10.3 |
| Interquartile ranges (IQR) |  | 7.9-14.9 |
| **Albumin level at week-6** | **NI** |  |
| Median |  | 39.6 |
| Interquartile ranges (IQR) |  | 37.3-43.0 |
| **LDH at week-6** | **NI** |  |
| Median |  | 168..3 |
| Interquartile ranges (IQR) |  | 144.9-194-5 |
| **CEA at week-6** | **NI** |  |
| Median |  | 6.57 |
| Interquartile ranges (IQR) |  | 3.94-17.8 |
| **CA199 at week-6** | **NI** |  |
| Median |  | 240.2 |
| Interquartile ranges (IQR) |  | 34.6-1380 |
| **Bilirubin at week-6** | **NI** |  |
| Median |  | 10.3 |
| Interquartile ranges (IQR) |  | 7.9-14.9 |

**TableS5.** Survival analysis of first-line chemotherapy regimens in train group

| First-line chemotherapy regimens | N | mOS (months) | HR | 95%CI | |
| --- | --- | --- | --- | --- | --- |
| Gemcitabine monotherapy | 31 | 6.18 | 1 | 1 | 1 |
| nab-paclitaxel plus S1 | 134 | 9.92 | 1.044 | .476 | 2.292 |
| Gemcitabine-based combination | 41 | 11.04 | .580 | .281 | 1.196 |
| nab-paclitaxel plus gemcitabine | 16 | 10.12 | .673 | .307 | 1.474 |
| Others | 11 | 4.27 | .582 | .230 | 1.477 |
| Total | 233 | 9.56 |  |  |  |

**Table S6.** Results of univariate survival analysis in train cohort (supplement data)

| **Characteristic** | **Baseline model**  **(N=323)** | | | **Chemotherapy response-based model (N=233)** | | |
| --- | --- | --- | --- | --- | --- | --- |
|  | HR | 95% CI | *p* value | HR | 95% CI | *p* value |
| **BMI(≤**medium vs＞medium**)** | 1.02 | 0.97-1.06 | 0.493 | 0.92 | 0.66-1.29 | 0.629 |
| **Alcohol** (No vs Yes) | 1.08 | 0.82-1.42 | 0.568 | 1.19 | 0.88-1.63 | 0.251 |
| **Diabetes** (No vs Yes) | 0.91 | 0.66-1.27 | 0.590 | 0.90 | 0.62-1.31 | 0.581 |
| **Objective jaundice** (No vs Yes) | 1.29 | 0.94-1.77 | 0.109 | 1.16 | 0.81-1.66 | 0.419 |
| **Primary site** (head vs body and tail) | 1.00 | 0.76-1.32 | 0.994 | 1.04 | 0.76-1.42 | 0.809 |
| **Number of organ metastases** |  |  |  |  |  |  |
| 0 |  | **Reference** |  |  | **Reference** |  |
| 1 | 1.34 | 0.92-1.95 | 0.122 |  |  |  |
| ≥2 | 1.27 | 0.83-1.95 | 0.266 |  |  |  |
| **Liver metastases** (absent vs present) | 1.29 | 0.94-1.78 | 0.112 | 1.19 | 0.83-1.72 | 0.339 |
| **Lung metastases** (absent vs present) | 0.88 | 0.62-1.25 | 0.481 | 1.08 | 0.74-1.59 | 0.683 |
| **Bone metastases** (absent vs present) | 1.07 | 0.68-1.68 | 0.750 | 1.11 | 0.65-1.89 | 0.704 |
| **Stage** (III vs IV) | 1.32 | 0.92-1.90 | 0.130 | 1.24 | 0.81-1.92 | 0.308 |
| **PLT** (≤medium vs＞medium) | 0.81 | 0.62-1.05 | 0.113 | 0.80 | 0.59-1.09 | 0.153 |
| **Change in Alb level at week 6** |  | **NI** |  |  |  |  |
| Decrease |  |  |  |  | Ref |  |
| No change or increase |  |  |  | 1.14 | 0.83-1.57 | 0.414 |
| **Change in LDH level at week 6** |  | **NI** |  |  |  |  |
| Decrease |  |  |  |  | Ref |  |
| No change or increase |  |  |  | 0.93 | 0.68-1.28 | 0.667 |
| **Change in TB level at week 6** |  | **NI** |  |  |  |  |
| Decrease |  |  |  |  | Ref |  |
| No change or increase |  |  |  | 1.06 | 0.78-1.45 | 0.669 |
| **LDH at week 6** |  | **NI** |  |  |  |  |
| Decrease |  |  |  |  | Ref |  |
| No change or increase |  |  |  | 1.04 | 0.77-1.40 | 0.813 |
| **Chemotherapy regimen** |  | **NI** |  |  |  |  |
| Non nab-paclitaxel plus S1 |  |  |  |  | Ref |  |
| nab-paclitaxel plus S1 |  |  |  | 0.73 | 0.54-0.99 | **0.040** |
| **Second-line chemotherapy** |  | **NI** |  |  |  |  |
| Yes |  |  |  |  | Ref |  |
| No |  |  |  | 1.63 | 1.19-2.20 | **0.002** |
| **Previous surgery** |  | **NI** |  |  |  |  |
| Yes |  |  |  |  | Ref |  |
| No |  |  |  | 1.34 | 0.68-2.64 | 0.385 |


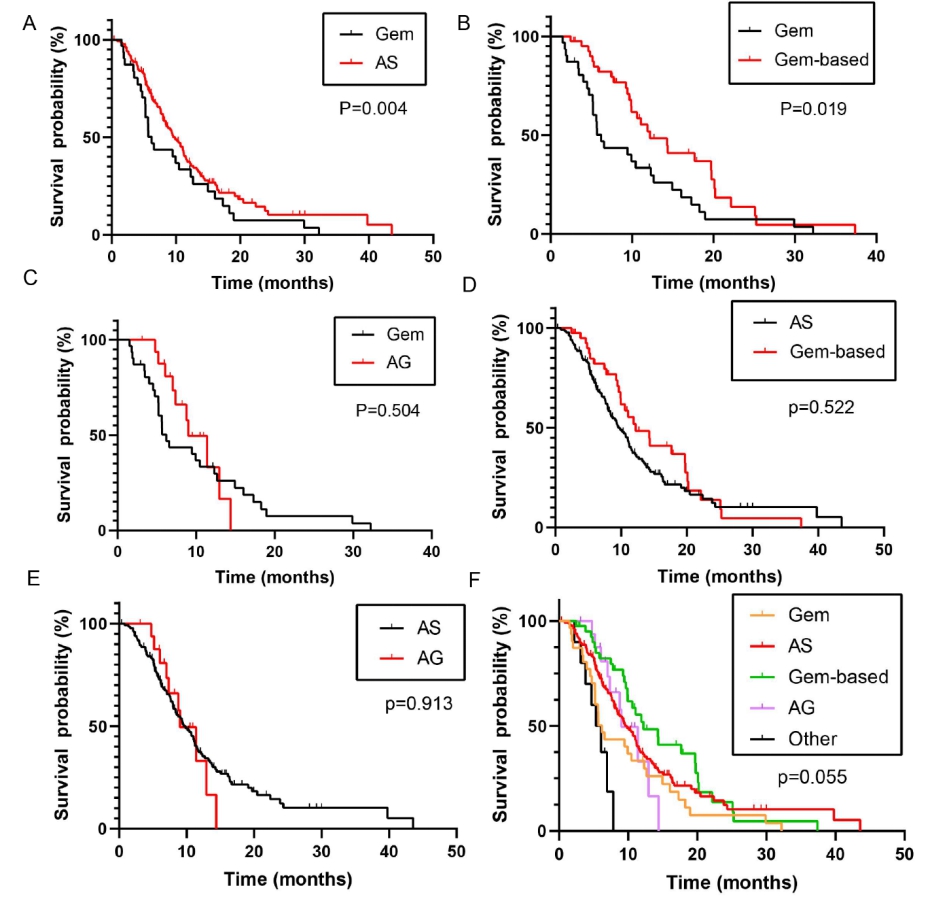


FigureS1. Kaplan-Meier survival curves of different chemotherapy regimens. A. Gem vs AS. B. Gem vs Gem-based. C. Gem vs AG. D. AS vs Gem-based. E. AS vs AG. F. Survival analysis of all chemotherapy regimens.

Abbreviation: Gem: gemcitabine monotherapy; Gem-based: gemcitabine-based combination chemotherapy; AS: nab-paclitaxel plus S1; AG: nab-paclitaxel plus gemcitabine
