# Supplementary material for: Nomogram to predict survival of patients with advanced and metastatic pancreatic Cancer
Source: BMC Cancer. 2021 Nov 15;21:1227. doi: 10.1186/s12885-021-08943-w (PMC8594118; doi:10.1186/s12885-021-08943-w)
Supplement: Supplementary file 1 — Additional file 1: Table S1. Patient characteristics of train groups (supplement data). Table S2. Patient Characteristics of train groups. Table S3. Patient characteristics of the validation groups. Table S4. Patient characteristics of validation groups. Table S5. Survival analysis of first-line chemotherapy regimens in train group. Table S6. Results of univariate survival analysis in train cohort (supplement data). Fig. S1. Kaplan-Meier survival curves of different chemotherapy regimens. A. Gem vs AS. B. Gem vs Gem-based. C. Gem vs AG. D. AS vs Gem-based. E. AS vs AG. F. Survival analysis of all chemotherapy regimens. Abbreviation: Gem: gemcitabine monotherapy; Gem-based: gemcitabine-based combination chemotherapy; AS: nab-paclitaxel plus S1; AG: nab-paclitaxel plus gemcitabine. Fig. S2. Correlation analysis between twelve survival-related variables in baseline group. Fig. S3. Correlation analysis between eighteen survival-related variables in chemotherapy group. [file 12885_2021_8943_MOESM1_ESM.zip › Additional figures1.doc]

FigureS1. Kaplan-Meier survival curves of different chemotherapy regimens. A. Gem vs AS. B. Gem vs Gem-based. C. Gem vs AG. D. AS vs Gem-based. E. AS vs AG. F. Survival analysis of all chemotherapy regimens.

Abbreviation: Gem: gemcitabine monotherapy; Gem-based: gemcitabine-based combination chemotherapy; AS: nab-paclitaxel plus S1; AG: nab-paclitaxel plus gemcitabine

FigureS2.Correlation analysis between twelve survival-related variables in baseline group

FigureS3.Correlation analysis between eighteen survival-related variables in chemotherapy group
